# Prostate-Specific Antigen Kinetics Effects on Outcomes of Low-Volume Metastatic Prostate Cancer Patients Receiving Androgen Deprivation Therapy

**DOI:** 10.1155/2021/9648579

**Published:** 2021-08-26

**Authors:** Yen-Chi Lin, Po-Hung Lin, I-Hung Shao, Yuan-Cheng Chu, Hung-Cheng Kan, Chung-Yi Liu, Kai-Jie Yu, Ying-Hsu Chang, See-Tong Pang, Jhen-Ling Huang, Cheng-Keng Chuang

**Affiliations:** ^1^Division of Urology, Department of Surgery, Chang Gung Memorial Hospital and Chang Gung University, Taoyuan, Taiwan; ^2^Division of Urology, Department of Surgery, New Taipei Municipal TuCheng Hospital, Chang Gung Memorial Hospital and Chang Gung University, New Taipei City, Taiwan; ^3^Center for Big Data Analytics and Statistics, Chang Gung Memorial Hospital, Taoyuan, Taiwan

## Abstract

**Background:**

The present study aimed to analyse factors influencing the effects of androgen deprivation therapy (ADT) in patients with newly diagnosed metastatic castration-naïve prostate cancer (mCNPC), especially in low-volume disease (LVD), according to subclassification of metastatic prostate cancer established by the CHAARTED trial.

**Materials and Methods:**

We reviewed 648 patients with newly diagnosed mCNPC receiving ADT at Chang Gung Memorial Hospital from January 2007 to December 2016. Basic characteristics and PSA kinetics profile were subsequently evaluated.

**Results:**

48.3% of LVD patients progressed to castration-resistant prostate cancer (mCRPC). Among them, CRPC group had significantly shorter time to PSA nadir (TTN) and faster time from PSA nadir to CRPC (TFNTC) (*p* < 0.001) compared to non-CRPC group. PSA doubling time (PSADT) < 4 months tended to be associated with faster disease progression and shorter overall survival (OS). Among all patients with metastatic prostate cancer, those with shorter TTN <9 months, higher nadir PSA level ≥1 ng/mL, and shorter PSADT <3 months had increased tendency for biochemical progression.

**Conclusions:**

PSADT is an effective clinical predictor for disease progression and survival in LVD. Other PSA kinetics including TTN and TFNTC, though not the major predictors for disease progression or OS in LVD, might be the predictors for disease control status.

## 1. Background

Prostate cancer is one of the most common malignancies worldwide and the fifth most common cancer in Taiwan, with an age-standardised prostate cancer rate of 31.65 per 100,000 individuals in 2017 and metastatic prostate cancer (mPCa) accounting for nearly 30% of new cases [1–4]. Androgen deprivation therapy (ADT) has been the gold standard for patients with mPCa. Unfortunately, after receiving ADT, most of the prostate cancer cells develop drug resistance and progress to castration-resistant prostate cancer (CRPC), necessitating chemotherapy or second-line hormone therapy if feasible. Recent clinical trials and research have shown that upfront chemotherapy plus ADT promoted significantly longer overall survival (OS) in high-volume disease (HVD) [5–8]. “Tumor volume,” a new subclassification of metastatic prostate cancer established by the CHAARTED trial, can be classified as “high-volume” (visceral metastases and/or four or more bone metastases with at least one outside the vertebral column and pelvis) or “low-volume.” Moreover, this study showed that patients with HVD receiving a combination of ADT and chemotherapy had a longer median OS (49.2 months) than those receiving ADT alone (32.2 months), but LVD did not have survival benefit receiving chemohormonal therapy [5].

Apart from tumor volume, prostate-specific antigen (PSA) has been recognised as an important biomarker for predicting treatment response and disease progression in prostate cancer during ADT. The PSA kinetics profile, including initial PSA level (iPSA) [9], time to PSA nadir (TTN) [9–12], nadir PSA level [9, 13, 14], PSA decline pattern [15–17], and PSA doubling time (PSADT) [18], had been shown to reflect tumor burden and predict outcomes in patients with prostate cancer under ADT. Other than PSA level and related factors, pretreatment parameters, including Gleason grade group, haemoglobin (Hb), and alkaline phosphatase (ALP), have also been identified as prognostic or predictive biomarkers in mCNPC patients [19].

Most previous studies had included populations comprising patients with metastatic castration-naïve prostate cancer (mCNPC) or metastatic castration-resistant prostate cancer (mCRPC). However, since the CHAARTED trial, the concept of tumor volume had been integrated into the management of metastatic prostate cancer. As such, identifying reliable predicting factors for disease progression or overall survival according to different tumor volumes during ADT would be helpful in the prompt formulation of better treatment strategies for patients with poor prognostic characteristics. Several studies have investigated factors influencing disease burden in HVD [19, 20], while few of them discussed the risk factors for disease burden in low-volume disease (LVD). Thus, the current study aimed to explore the risk factors, particularly PSA kinetics, associated with poor prognosis, including shorter OS and shorter time to CRPC (the duration from initiation of ADT to biochemical CRPC status) in LVD under ADT.

## 2. Materials and Methods

### 2.1. Study Design and Patient Selection

We retrospectively evaluated patients with newly diagnosed metastatic prostate cancer between January 2007 and December 2016 who had received primary ADT (either surgical or medical castration, with or without antiandrogen) at Chang Gung Memorial Hospital (CGMH) Linkou branch. Patients were grouped into HVD and LVD according to the CHAARTED trial. This study adhered to the tenets of the Declaration of Helsinki and was approved by the Institutional Review Board (IRB) of Chang Gung Medical Foundation (IRB number 201801377B0). Patient consent is not required for observational studies.

### 2.2. Clinical Data and Outcome Collection

CRPC was diagnosed based on biochemical progression (three consecutive spikes in PSA 1 week apart, of which two were 50% higher than the nadir, and PSA >2 ng/mL) according to the European Association of Urology guideline. OS was defined as the period from diagnosis until death by any cause. Baseline patient demographics and posttreatment characteristics, including age at diagnosis, clinical M staging, iPSA (PSA level upon diagnosis), Gleason grade group (according to the classification of International Society of Urological Pathology [21]: grade group 1, Gleason score ≤6; grade group 2, Gleason score 3 + 4 = 7; grade group 3, Gleason score 4 + 3 = 7; grade group 4, Gleason score 8; and grade group 5, Gleason score ≥9), initial haemoglobin (Hb), calcium (Ca), and alkaline phosphatase (ALP) level, were determined. PSA kinetics profiles were defined based on previous related studies [11, 19, 22, 23], including TTN (defined as the duration from ADT initiation to PSA nadir), nadir PSA level, 3 months' PSA reduction rate (defined as (iPSA, posttreatment 3 months' PSA)/iPSA), PSA reduction rate (PSARR) (defined as 100 × (iPSA − PSA nadir/iPSA)/TTN) [24], time to CRPC (the duration from ADT initiation to biochemical CRPC status), time from PSA nadir to CRPC (TFNTC) (the duration from PSA nadir to CRPC status), and PSADT (defined as log2 × (time interval)/log (PSA value)-log (nadir PSA)). Patients with insufficient imaging reports for determining volume status or excessive missing data were excluded.

Previous studies have demonstrated that several cutoff points for PSA-related factors, including iPSA, TTN, PSA nadir, and PSADT, predicted disease progression or OS [10, 11, 13, 24, 25]. Moreover, some studies have utilised the receiver operating characteristic curve to determine cutoff points, while others use median values. The current study chose median values as the optimal cutoff points for the different parameters.

### 2.3. Statistical Analysis

All statistical analyses were performed using SAS software version 9.4 (SAS Institute Inc., Cary, NC). Nominal variables are presented as means and standard deviations, while nonnominal variables are presented as medians and interquartile ranges. The Chi-square test was used to compare categorical variables, while the independent *t*-test was used to compare continuous variables. OS and CPRC-free survival were evaluated using the Kaplan–Meier method. The multivariate Cox proportional-hazards model was used to determine the association between risk factors and OS or CPRC-free survival. All *p* values reported were two sided with *p* < 0.05 indicating statistical significance.

## 3. Results

### 3.1. Characteristics of the Study Population

**Figure 1:**
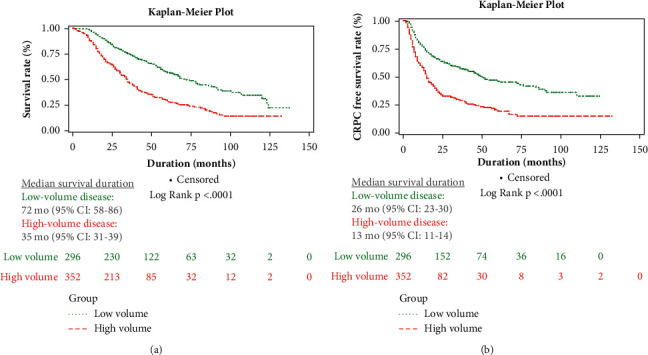
(a) Kaplan–Meier plot of overall survival with high-volume disease and low-volume disease. (b) Kaplan–Meier plot of CRPC free survival between patients with high-volume disease and low-volume disease. CI: confidence interval.

A total of 918 patients with newly diagnosed metastatic prostate cancer at CGMH from January 2007 to December 2016 were identified. After excluding those who had received either chemotherapy or radiotherapy and those with incomplete information, a total of 648 patients receiving primary ADT were ultimately analysed. In the study population (Table 1), included patients had a median age of 75 (IQR 68–80) years, with 352 (54.3%) classified as HVD and 296 (45.7%) as LVD according to the CHAARTED trial. At the median follow-up of 34 months (range from 1 month to 137 months), a total of 371 (57%) patients died during the study period. The median OS was 48 months (Table 2), while those with HVD had a significantly shorter median OS than those with LVD (35 vs. 72 months; *p* < 0.0001) (Figure 1(a)).

### 3.2. Comparison between High-Volume Disease and Low-Volume Disease

A total of 375 (57.9%) patients progressed to biochemical CRPC status (Table 2); among them, 232 (61.9%) patients were HVD and 143 (38.1%) were LVD. The median time to CRPC in all patients, those with HVD, and those with LVD was 16.5, 13, and 26 months, respectively (*p* < 0.0001) (Figure 1(b)). Significant differences in baseline characteristics and PSA kinetics, including Hb, ALP, iPSA, Gleason grade group, TTN, PSARR, PSA nadir level, TFNTC, and PSADT, were observed between patients with HVD and LVD (Table 1). In the subgroup analysis, patients with LVD were divided into CRPC group (48.3%) and non-CRPC group (51.7%). Among them, CRPC group had similar nadir PSA level, 3-month PSA reduction rate, and PSADT, but significantly shorter TTN (8.7 vs. 16.7 months) and faster TFNTC (4.3 vs. 15.6 months) (*p* < 0.001) compared to the non-CRPC group (Table 3).

### 3.3. Affecting Factors for Overall Survival and Disease Progression

Multivariate analysis of all included patients (mCNPC) revealed that those with TTN <9 months, nadir PSA level ≥1 ng/mL, and PSADT <3 months had increased tendency for biochemical progression (Table 4), while those with TTN <9 months, nadir PSA level ≥1 ng/mL, duration of PSA nadir <5 months, PSADT <3 months, and time to CRPC <17 months had increased risk for shorter OS (Table 5). Univariate analysis of patients with LVD showed that Gleason grade group and PSA kinetics including iPSA, PSARR, TFNTC, TTN, and PSADT were statistically significant parameters for disease progression and OS; however, multivariate analysis of patients with LVD showed that only PSADT (<4 months) was associated with increased risk for early disease progression to CRPC and shorter OS (Tables 6 and 7).

## 4. Discussion

Research has shown that upfront chemotherapy with first-line ADT significantly improved OS in patients with high-volume mCNPC [5]. Accordingly, previous studies had identified age, ECOG performance status, Gleason grade group, pretreatment Hb, ALP, LDH, nadir PSA level, TTN, and PSADT as prognostic factors for mPCa [9, 19, 22]. Apart from the aforementioned factors, Guangjie et al. demonstrated that patients who exhibited a rapid decrease in PSA levels during the initial ADT phase were at increased risk for progression to CRPC [16]. Nakayama et al. introduced the concept that PSA kinetics and early PSA decline were associated with time to PSA progression differently in patients with mCRPC receiving abiraterone acetate. However, the aforementioned study had a limited number of patients and events [26]. Accordingly, the present study found that PSA kinetics was strongly associated with either risk for disease progression or OS. To date, three major clinical trials, namely, GETUG-AFU 15, CHAARTED, and STAMPEDE, had incorporated early chemohormonal therapy into their treatment stagey for mCNPC [27]. Among the aforementioned trials, only CHAARTED had classified the study groups based on tumor volume, subsequently demonstrating a statistically significant improvement in OS for patients with HVD on chemohormonal therapy [27].

HVD accounted for 54% of our study population, 63% of that in the CHARTEED trial, and 49% of that in previous studies within Taiwan [20]. The present study found that patients on ADT with HVD had significantly shorter OS (30 vs. 43 months) and time to the development of CRPC (13 vs. 26 months) compared to those with LVD. This agrees with the general consensus that HVD promotes worse prognosis due to disease severity.

PSA level has been the most widely used biomarker for evaluating disease progression and predicting survival in clinical practice. Furthermore, several retrospective clinical studies and even some meta-analyses have determined that PSA kinetics, including PSA response rate, nadir PSA level, TTN, or PSADT, predicted OS or disease progression [9, 10, 13, 24]. PSADT predicting outcomes has been known for nearly 30 years, and it may closely indicate changes in prostate tumor volume, an independent predictor of biochemical relapse among patients that either underwent radical prostatectomy or endocrine treatment [28]. Doctor D'Amico et al. demonstrated that patients with clinically localized prostate cancer treated with external beam radiation therapy with PSADT >12 months have lower risk of prostate cancer death within 5 years of relapse [29]. Moreover, Kelloff et al. reported that PSADT was a predictor of tumor response to medication in patients with prostate cancer and suggested that significant changes in PSADT may be used to support the approval of newer treatment [30]. Despite the considerably wide distribution of PSADT values, studies have suggested that it may still be useful for strategies after relapse and that patients with advanced or relapsed disease who have rapid PSADT should receive more aggressive or earlier treatment [31, 32]. Tomioka et al. also reported that posttreatment PSADT ≤2 months may be a predictor for decreased survival and was associated with poor prognosis among patients with bone metastatic prostate cancer [33]. The present study identified lower cutoff point of PSADT than before as a useful clinical predictor in patients with LVD receiving ADT (PSADT <4 months). The possible cause of short median PSADT in our study is that the PSA nadir level is low, so that the doubled PSA level could be reached easily. In addition, PSA level is checked more frequently in our study than in other studies, so the PSADT may also be influenced. Although PSADT cannot be evaluated during the early stages of ADT, it may still be a clinically effective predictor of disease progression and OS.

Previous reports have shown that nadir PSA levels, TTN, and even PSA reduction pattern have been considered important predictors of survival and progression period. Generally, a faster decline in PSA levels has been associated with more cancer cell death, which promotes a more favourable prognosis and survival [34]. However, Ji et al. reported in their retrospective study that rapidly decreasing PSA levels during initial ADT was a risk factor for early progression to CRPC [16]. Accordingly, a recent study introduced the novel concept suggesting that tumor-regulating fibroblasts play an important role in the mechanisms associated with TTN after primary ADT [35]. Other studies have also demonstrated that rapidly decreasing PSA levels may be associated with transcriptional outcomes of ADT rather than cancer cell death. Moreover, heterogeneous prostate cancer cells, including hormone-resistant prostate cancer cells and hormone-sensitive prostate cancer cells, often coexist in the same patient. The rapid decline in PSA levels may indicate downregulation of PSA expression in hormone-sensitive prostate cancer cells, which are regulated by androgen via the androgen receptor pathway [36]. Although it is doubtful that whether hormone-sensitive prostate cancer cells account for more of the cancer cells in HVD than in LVD, it might be a possible explanation for patient with HVD to have shorter TTN and faster time to disease progression, even shorter OS. Moreover, longer TTN and lower PSA nadir levels during ADT have been known to be associated with significantly longer OS and period of disease progression [10, 11, 13, 24, 37]. Hamano et al. demonstrated that higher PSA nadir and shorter TTN are poor prognostic factors in men with mCRPC on ADT [24], and we might presume that TTN and PSA nadir are the effective predictors for disease progression in relative advanced prostate cancer. Given that LVD has been associated with a lower CRPC rate and less severe bone metastases and disease status, TTN, PSA nadir, and PSA decline pattern might not be effective predictors for OS and disease progression in LVD. Notably, PSA response rates (even 4 weeks, 12 weeks, or maximal response rate) were used to evaluate the treatment response for patients with prostate cancer in previous studies [38, 39], so in LVD, even though PSA decline rate is not a major predictor for OS or disease progression in the present study, it might still have the role in treatment response evaluation.

The major implication of the current study is that, among patients with LVD, more attention should be provided to those with increasing PSA levels and PSADT considering that TTN, PSA nadir, and even PSA reduction pattern were not major predictors for faster disease progression or OS. To our knowledge, early chemotherapy or androgen metabolism inhibitors (abiraterone with prednisone) therapy is not recommended in LVD initially, but some of them might be suitable for early usage of second-generation antiandrogen such as apalutamide and enzalutamide. In the present study, disease that progressed to CRPC was noted in 143 (48.3%) patients with LVD, and we found that they had similar nadir PSA level, 3-month PSA reduction rate, and PSADT, but significantly faster TTN (8.7 vs. 16.7 months) and faster TFNTC (4.3 vs. 15.6 months) (*p* < 0.001) compared to patients with no CRPC in LVD. In addition, in univariate analysis, TTN and TFNTC were also statistically significant parameters for disease progression. Even though in multivariate analysis, only PSADT is shown to be predictor for disease progression, we can still hypothesize that patient with faster TTN and shorter TFNTC might be related to disease that progressed to CRPC in LVD. The possible cause is that TTN and TFNTC are significantly different in patient with CRPC or not, so the cutoff point we chose might not be the optimal cutoff point for analysis. Even though TTN and TFNTC are not the major predictors for disease progression in LVD, they might still be the parameters for disease control evaluation. Patients with faster TTN and shorter TFNTC might be the hints, even the predictors for poor disease control status for us so that we could early apply chemotherapy or the new generation hormone therapy such as abiraterone or enzalutamide for better disease control or quality of life improvement.

## 5. Limitations

The current study has some limitations mentioned as follows. First, the most important limitation is our exclusion of patients who received chemotherapy or radiotherapy; however, those with subsequent hormone therapy, including abiraterone or enzalutamide after CRPC, were not excluded, which may have affected our results with regard to OS. Second, given that this was a single-centre retrospective study, our results may not be generalisable to other populations. Moreover, considering that Linkou CGMH is a medical centre in northern Taiwan, the disease severities of our study population may be higher than those of general population. Lastly, not all PSA parameters and factors, including Gleason score, Hb, Ca, and ALP, were regularly assessed at our institution, which might have influenced our results.

## 6. Conclusion

Among patients receiving ADT, those with high-volume metastatic prostate cancer had significantly shorter OS and faster disease progression compared to those with low-volume metastatic prostate cancer. Moreover, the current study demonstrated that PSADT ≥4 months had been identified as an effective predictor for slower disease progression and better OS in LVD. Furthermore, other PSA kinetics including TTN, PSA decline pattern, and PSA nadir were not the major predictors for disease progression or OS in LVD, but TTN and time from PSA nadir to CRPC (TFNTC) might be the predictors for disease control status.

## Figures and Tables

**Table 1:** Baseline characteristics.

Patient demographics	HVD (*n* = 352, 54.3%)	LVD (*n* = 296, 45.7%)	*p* value
Age, median, year (IQR)	75 (68–80)	74 (68–80)	0.2242
M stage, *n* (%)			<0.0001
1A	0	72 (24.3%)	
1B	268 (76.1%)	224 (75.7%)	
1C	84 (23.9%)	0	
Gleason grade group, *n* (%)			0.0013
1	0	8 (3.3%)	
2	6 (2.1%)	12 (4.9%)	
3	26 (9.1%)	34 (13.9%)	
4	64 (22.3%)	47 (19%)	
5	191 (66.5%)	145 (58.9%)	
Initial PSA level, ng/ml, median (IQR)	511.5 (181.25–1234.47)	98.2 (45–256.6)	<0.0001
Hb, g/dL, median (IQR)	11.5 (9.7–13.2)	12.7 (11.6–13.9)	<0.0001
Ca, g/dL, median (IQR)	8.7 (8.2–9.1)	8.8 (8.3–9.1)	0.9528
ALP, U/L, median (IQR)	146 (94–324)	74 (60–101)	<0.0001
CRPC, *n* (%)			<0.0001
Yes	232 (65.9%)	143 (48.3%)	
No	120 (34.1%)	153 (51.7%)	
PSA kinetics after ADT			
TTN, month, median (IQR)	7.15 (3.7–13.3)	11.9 (6.25–20.9)	<0.0001
Nadir PSA level, ng/ml, median (IQR)	2.2 (0.23–17.18)	0.23 (0.02–1.7)	0.0015
PSARR, %/month, median (IQR)	12.5 (6.8–22.04)	7.99 (4.38–14.9)	0.0002
TFNTC, month, median (IQR)	3.25 (2.5–7.9)	8.3 (2.7–26.1)	<0.0001
PSADT, month, median (IQR)	2.3 (1.4–4.15)	3.6 (1.9–7)	<0.0001

CRPC: castration-resistant prostate cancer, TTN: time to PSA nadir, PSARR: PSA reduction rate, TFNTC: time from PSA nadir to CRPC, and PSADT: PSA doubling time.

**Table 2:** Overall baseline characteristics.

Patient demographics	All patients (*n* = 648)
Age, median, year (IQR)	75 (68–80)
Overall survival, median, month (IQR)	48 (24–106)
Time to CRPC, median, month (IQR)	16.5 (8–36)
M stage, *n* (%)	
1A	72 (11.11%)
1B	492 (75.93%)
1C	84 (12.96%)
Gleason grade group, *n* (%)	
1	8 (1.5%)
2	18 (3.38%)
3	60 (11.26%)
4	111 (20.83%)
5	336 (63.04%)
Missing	115
Initial PSA level, ng/ml, median (IQR)	243.9 (74.9–790.8)
Hb, g/dL, median (IQR)	12.1 (10.3–13.5)
Ca, g/dL, median (IQR)	8.7 (8.3–9.1)
ALP, U/L, median (IQR)	118 (75–227)
CRPC, *n* (%)	
Yes	375 (57.9%)
No	273 (42.1%)
PSA kinetics after ADT	
TTN, month, median (IQR)	8.7 (4.8–16.65)
Nadir PSA level, ng/ml, median (IQR)	0.86 (0.07–5.99)
PSARR, %/month, median (IQR)	10.32 (5.62–18.17)
TFNTC, month, median (IQR)	4.5 (2.7–15.1)
PSADT, month, median (IQR)	2.6 (1.5–5.3)

CRPC: castration-resistant prostate cancer, TTN: time to PSA nadir, PSARR: PSA reduction rate, TFNTC: time from PSA nadir to CRPC, and PSADT: PSA doubling time.

**Table 3:** PSA kinetics after ADT in LVD.

	CRPC (143, 48.3%)	Non-CRPC (153, 51.7%)	
TTN, month, median (IQR)	8.7 (5.4–14.1)	16.7 (8.7–26.6)	<0.0001
Nadir PSA level, ng/ml, median (IQR)	0.75 (0.12–2.92)	0.04 (0.01–0.41)	0.618
3-month PSARR, %, median (IQR)	95.64 (87.67–98.54)	97.18 (92.65–99.16)	0.214
TFNTC, month, median (IQR)	4.3 (2.7–11.65)	15.6 (4–41.1)	<0.0001
PSADT, month, median (IQR)	3.65 (1.9–6.875)	3.4 (1.95–7.325)	0.534

CRPC: castration-resistant prostate cancer, TTN: time to PSA nadir, 3-month PSARR: 3-month PSA reduction rate, TFNTC: time from PSA nadir to CRPC, and PSADT: PSA doubling time.

**Table 4:** Univariate and multivariate analysis of predicting factors for time to CRPC.

Factors	Univariate hazard ratio (95% CI)	*p* value	Multivariate hazard ratio (95% CI)	*p* value
Age				
<75	1 (reference)			
≥75	0.97 (0.79–1.18)	0.731	—	
Gleason grade group				
<4	1 (reference)			
≥4	1.75 (1.25–2.46)	0.001	—	
Initial PSA level, ng/ml				
<250	1 (reference)			
≥250	1.69 (1.34–2.14)	<0.0001	—	
TTN, month				
<9	1 (reference)		1 (reference)	
≥9	0.27 (0.22–0.33)	<0.0001	0.22 (0.11–0.44)	<0.0001
Nadir PSA level, ng/ml				
<1	1 (reference)		1 (reference)	
≥1	3.78 (2.93–4.88)	<0.0001	3.11 (2.25–4.29)	<0.0001
PSARR, % (month)				
<10	1 (reference)		1 (reference)	
≥10	3.76 (2.93–4.81)	<0.0001	0.59 (0.31–1.15)	0.123
TFNTC, month				
<5	1 (reference)		1 (reference)	
≥5	0.19 (0.15–0.24)	<0.0001	1.37 (0.99–1.89)	0.059
PSADT, month				
<3	1 (reference)		1 (reference)	
≥3	0.34 (0.26–0.44)	<0.0001	0.34 (0.25–0.48)	<0.0001

TTN: time to PSA nadir, PSARR: PSA reduction rate, TFNTC: time from PSA nadir to CRPC, and PSADT: PSA doubling time.

**Table 5:** Univariate and multivariate analysis of predicting factors for OS.

Factors	Univariate hazard ratio (95% CI)	*p* value	Multivariate hazard ratio (95% CI)	*p* value
Age				
<75	1 (reference)			
≥75	1.37 (0.11–1.68)	<0.001	—	
Gleason grade group				
<4	1 (reference)			
≥4	1.82 (1.29–2.59)	<0.0001	—	
Initial PSA level, ng/ml				
<250	1 (reference)			
≥250	1.29 (1.03–1.62)	0.030	—	
TTN, month				
<9	1 (reference)		1 (reference)	
≥9	0.25 (0.20–0.31)	<0.0001	0.24 (0.12–0.48)	<0.0001
Nadir PSA level, ng/ml				
<1	1 (reference)		1 (reference)	
≥1	3.66 (2.86–4.68)	<0.0001	2.76 (1.98–3.84)	<0.0001
PSARR, % (month)				
<10	1 (reference)		1 (reference)	
≥10	3.51 (2.73–4.51)	<0.0001	0.53 (0.27–1.04)	0.065
TFNTC, month				
<5	1 (reference)		1 (reference)	
≥5	0.38 (0.30–0.47)	<0.0001	1.67 (1.18–2.37)	0.004
PSADT, month				
<3	1 (reference)		1 (reference)	
≥3	0.25 (0.19–0.34)	<0.0001	0.40 (0.28–0.56)	<0.0001
TTC, month				
<17	1 (reference)		1 (reference)	
≥17	0.20 (0.16–0.25)	<0.0001	0.53 (0.32–0.85)	0.009

TTN: time to PSA nadir, PSARR: PSA reduction rate, TFNTC: time from PSA nadir to CRPC, PSADT: PSA doubling time, and TTC: time from ADT to CRPC.

**Table 6:** Univariate and multivariate analysis of predicting factors for time to CRPC in LVD.

Factors	Univariate hazard ratio (95% CI)	*p* value	Multivariate hazard ratio (95% CI)	*p* value
Age				
<75	1 (reference)			
≥75	0.94 (0.67–1.30)	0.699	—	
Gleason grade group				
<4	1 (reference)			
≥4	1.82 (1.13–2.96)	0.015	—	
Initial PSA level, ng/ml				
<100	1 (reference)			
≥100	1.03 (0.71–1.51)	0.864	—	
TTN, month				
<12	1 (reference)		1 (reference)	
≥12	0.25 (0.18–0.36)	<0.0001	0.31 (0.05–2.01)	0.222
Nadir PSA level, ng/ml				
<0.2	1 (reference)		1 (reference)	
≥0.2	5.61 (3.62–8.69)	<0.0001	2.09 (0.88–4.94)	0.094
3-mon PSARR, %				
<97				
≥97	1 (reference)			
1.49(0.98–2.26)	0.063			
PSARR, % (month)				
<8	1 (reference)		1 (reference)	
≥8	4.20 (2.79–6.35)	<0.0001	0.79 (0.13–4.86)	0.802
TFNTC, month				
<8	1 (reference)		1 (reference)	
≥8	0.18 (0.12–0.26)	<0.0001	0.97 (0.38–2.46)	0.944
PSADT, month				
<4	1 (reference)		1 (reference)	
≥4	0.45 (0.30–0.68)	<0.0001	0.31 (0.16–0.61)	0.001

TTN: time to PSA nadir, 3-mon PSARR: posttreatment 3 months' PSA reduction rate, PSARR: PSA reduction rate, TFNTC: time from PSA nadir to CRPC, and PSADT: PSA doubling time.

**Table 7:** Univariate and multivariate analysis of predicting factors for OS in LVD.

Factors	Univariate hazard ratio (95% CI)	*p* value	Multivariate hazard ratio (95% CI)	*p* value
Age				
<75	1 (reference)			
≥75	1.64 (1.15–2.32)	0.006	—	
Gleason grade group				
<4	1 (reference)			
≥4	1.71 (1.04–2.82)	0.034	—	
Initial PSA level, ng/ml				
<100	1 (reference)			
≥100	1.000.68–1.48)	0.995	—	
TTN, month				
<12	1 (reference)		1 (reference)	
≥12	0.30 (0.21–0.44)	<0.0001	0.32 (0.05–1.93)	0.214
Nadir PSA level, ng/ml				
<0.2	1 (reference)		1 (reference)	
≥0.2	3.37 (2.20–5.17)	<0.0001	1.87 (0.78–4.49)	0.159
3-mon PSARR, %				
<97	1	0.108		
≥97	1.44 (0.92–2.24)			
PSARR, % (month)				
<8	1 (reference)		1 (reference)	
≥8	3.99 (2.58–6.19)	<0.0001	0.71 (0.12–4.13)	0.703
TFNTC, month				
<8	1 (reference)		1 (reference)	
≥8	0.28 (0.19–0.40)	<0.0001	1.21 (0.46–3.19)	0.706
PSADT, month				
<4	1 (reference)		1 (reference)	
≥4	0.19 (0.11–0.32)	<0.0001	0.35 (0.17–0.71)	0.004
TTC, month				
<26	1 (reference)		1 (reference)	
≥26	0.18 (0.12–0.26)	<0.0001	0.57 (0.20–1.64)	0.298

TTN: time to PSA nadir, 3-mon PSARR: posttreatment 3 months' PSA reduction rate, PSARR: PSA reduction rate, TFNTC: time from PSA nadir to CRPC, PSADT: PSA doubling time, and TTC: time from ADT to CRPC.

## Data Availability

This study is based in part on data from the Chang Gung Research Database provided by Chang Gung Memorial Hospital. The interpretation and conclusions contained herein do not represent the position of Chang Gung Memorial Hospital.

## Abbreviations

mPCa: Metastatic prostate cancer
ADT: Androgen deprivation therapy
CRPC: Castration-resistant prostate cancer
OS: Overall survival
HVD: High-volume disease
PSA: Prostate-specific antigen
iPSA: Initial PSA level
TTN: Time to PSA nadir
PSADT: PSA doubling time
Hb: Haemoglobin
ALP: Alkaline phosphatase
mCNPC: Metastatic castration-naïve prostate cancer
mCRPC: Metastatic castration-resistant prostate cancer
LVD: Low-volume disease
Ca: Calcium
PSARR: PSA reduction rate
TFNTC: Time from PSA nadir to CRPC.
